# Identification of potential transcriptionally active Copia LTR retrotransposons in *Eucalyptus*

**DOI:** 10.1186/1753-6561-5-S7-P164

**Published:** 2011-09-13

**Authors:** Helena Marcon, Douglas Domingues, Celso Marino

**Affiliations:** 1 Departamento de Genética, UNESP, Botucatu, São Paulo, Brazil; 2 IAPAR, Londrina, Paraná, Brazil

## Background

Long Terminal Repeat retrotransposons (LTR-RTs) represent the most abundant genomic component in all plant genomes thus far investigated. They are transposable elements that replicate through a “copy/paste” mechanism that relies on reverse transcription and integration of a RNA intermediate. Plant LTR-RTs can be divided in two major superfamilies: *Copia* and *Gypsy*[1]. LTR-RTs have impact on genome size variation, as well as in the expression of adjacent genes in their host genomes, providing a “genomic plasticity” [2]. Their transcription was believed to be extremely repressed in plants. However, despite their potential mutagenic and deleterious effects, LTR-RTs were proven to be transcriptionally active in several plant species [3].

*Eucalyptus* is one of the most commercially important forest genus in the world, due to their superior growth, broad adaptability and multipurpose wood properties. Most molecular studies in *Eucalyptus* are focused on cellulose production and wood development, and there are few works on genome composition, structure and evolution. *Pinus* and *Populus*, the tree genera with most available genomic resources, have several works analyzing their repertoire of LTR-RTs [i. e 4, 5], but only one study characterized LTR-RTs in *Eucalyptus*[6], with no detailed manual checking or phylogenetic analysis. Here, we used FOREST database as a starting point to identify transcriptionally active *Copia* LTR-RTs in *Eucalyptus*, that were further analyzed regarding their *in silico* expression, evolutionary diversity, and distribution in public genomic databases.

## Methods

A previous survey with 88 *Copia*LTR-RTs from diverse plants defined six major common evolutionary *Copia*lineages [7]. The 22 *Arabidopsis thaliana* families analyzed in that study were used as queries to the identify *Eucalyptus*EST sequences related to *Copia*elements in FORESTS database [8], by tBLASTx (e-value >1e-50). Sequences were then analyzed in RepBase [9] to confirm their similarity to *Copia* LTR-RTs. *Eucalyptus*ESTs with >200bp of *copia*-like retrotransposon fragments were used to identify complete copies in *Eucalyptusgrandis* genome v 1.0 in a BLASTn search (identity >80%; in a region >250bp). We picked up 10000bp surrounding the aligned region, that were analyzed using LTR-Finder [10] and LTR_STRUC [11]. Full-length LTR-RTs were then used as queries in GenBank to retrieve related *Eucalyptus*EST sequences (>200bp; >80% identity). Phylogenetic analyses using the reverse transcriptase of these elements (alignment in MUSCLE, Maximimum Likelihood method, bootstrap 1000 replicates) were done using MEGA 5.01 [12].

## Results

**Figure 1:**
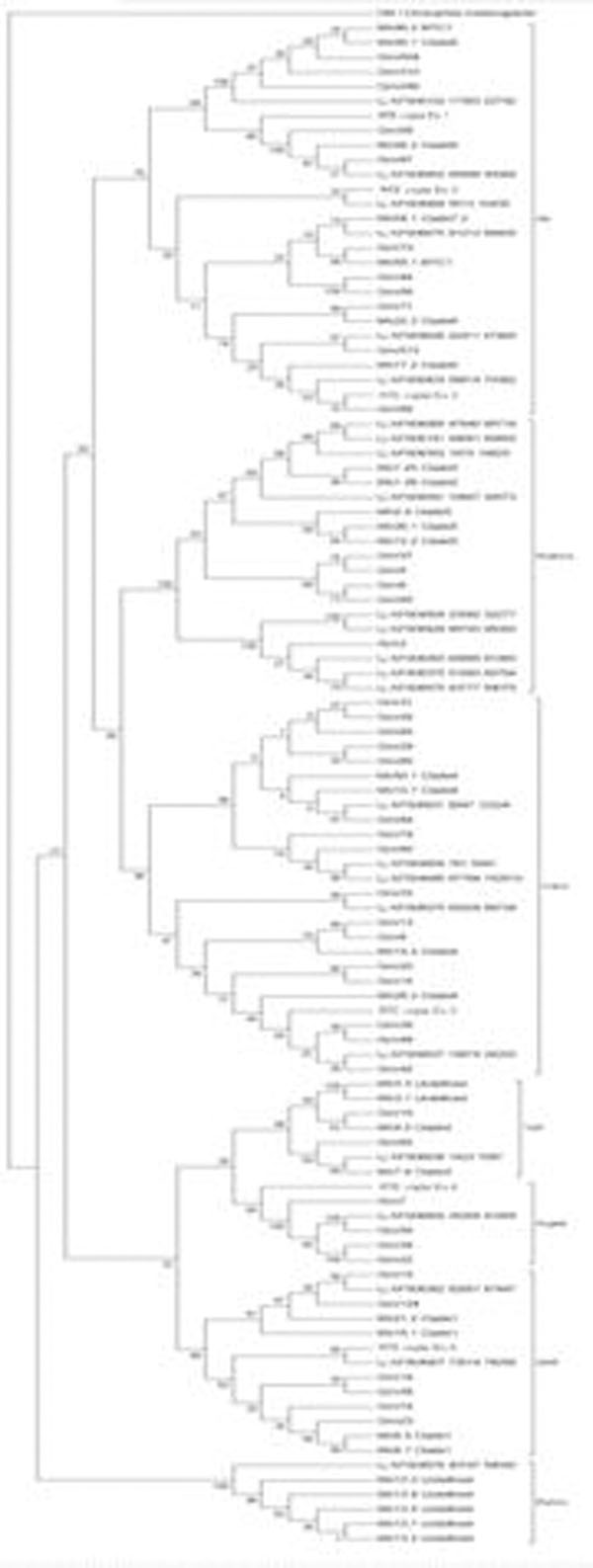
Phylogenetic tree of Copia LTR-RTs elements. Based on Du and collaborators (2010).

Stem, calli and seedlings were the cDNA libraries from FOREST database with most EST sequences, in this *Copia* LTR-RT search. We identified 20 consensus sequences (total: 36 ESTs) from 3 tissues, roots, leaves and flower-buds. We also identified 29 ESTs in GenBank from xylem, root apex and cold-stressed plants (Table 1). Using EST data, we identified six full-length retrotransposons families that had different copy number in the *Eucalyptus* genome, estimated by BLAST searches (cutoff 1e-50). Copy number ranged from 24 to 262 (Table 1). Phylogenetic analyses showed that they are members of the *Ale*, *Angela*, *GMR* and *Ivana* evolutionary lineages (figure 1). *Ale* was the evolutionary lineage encompassing families with highest and lowest copy number (Table 1).

**Table 1:** Overall features of LTR-RTs analyzed.

Family	Lineage	Genomic copy number	FOREST cDNA libraries	GenBank cDNA libraries
RTE_*copia*_Eu_ 1	*Ale*	28	seedlings	xylem
RTE_*copia*_Eu_ 2	*Ale*	262	roots, leaves	xylem
RTE_*copia*_Eu_ 3	*Ale*	24	root	xylem, cold-stressed
RTE_*copia*_Eu_ 4	*Angela*	243	seedlings, calli	xylem
RTE_*copia*_Eu_ 5	*Ivana*	54	leaves, root, calli, wood	xylem
RTE_*copia*_Eu_ 6	*GMR*	63	leaves, seedlings	xylem

## Conclusion

In summary, the present data demonstrate the potential impact of future studies about functional and genomic analysis of LTR-RTs in *Eucalyptus*. This is the first characterization of full-length *Copia* LTR-RTs families in *Eucalyptus* genome with potential transcriptional activity, giving insights about phylogenetic diversity and copy number variation of retrotransposons in this tree.
